# A rare case of perineal hamartoma associated with cryptorchidism and imperforate anus: case report

**DOI:** 10.1590/S1679-45082014RC2746

**Published:** 2014

**Authors:** Kleiton Gabriel Ribeiro Yamaçake, Amilcar Martins Giron, Uenis Tannuri, Miguel Srougi

**Affiliations:** 1 Division of Urology, Hospital das Clínicas, Universidade de São Paulo, São Paulo, SP, Brazil; 2 Division of Pediatrics Surgery, Hospital das Clínicas, Universidade de São Paulo, São Paulo, SP, Brazil

**Keywords:** Hamartoma, Anus, imperforate, Criptochidism, Perineum, Infant, newborn, Case reports

## Abstract

A full-term male neonate with anorectal anomaly and external perineal anomalies was referred to our service. Physical examination showed an epithelized perineal mass with cutaneous orifices, which had urine fistulization, hipotrofic perineal musculature, bilateral congenital clubfoot, hipospadic urethra, criptorquidy bilateral with nonpalpable testis and imperforate anus. A colostomy was constructed immediately after birth. The child underwent excision of perineal mass, bilateral orchidopexy, Duplay neourethroplasty and coloanal anastomosis at 3 months of age. The histopathological examination of the perineal mass revealed a hamartoma.

## RESUMO

Recém-nascido a termo do sexo masculino encaminhado ao nosso serviço por anomalia anorretal e anomalias perineais externas. O exame físico revelou massa perineal epitelizada, com orifícios cutâneos que apresentavam saída de urina, musculatura perineal hipotrófica, pé torto congênito bilateral, uretra hipospádica, criptorquidia bilateral com testículos não palpáveis e ânus imperfurado. Logo após o nascimento, o paciente foi submetido à colostomia. Aos 3 meses de idade, a criança foi submetida à excisão da massa perineal, orquidopexia bilateral, neouretroplastia a Duplay e anastomose coloanal. A análise anatomopatológica da massa perineal indicou hamartoma.

## INTRODUCTION

Neonates presenting perineal masses are uncommon. When encountered, most perineal masses are anorectal malformations, sacrococcygeal teratomas, rectal prolapse, or duplication cysts.^(1)^ We report here a case of an otherwise healthy newborn with anorectal anomaly and a perineal mass. Initially, the mass was believed to be a sacrococcygeal teratoma. The patient underwent mass excision and the histopathologic evaluation revealed a benign hamartoma. This case is presented because of its rarity.

## CASE REPORT

A full-term male neonate with anorectal anomaly and external perineal anomalies was referred to our service. Antenatal ultrasound at a gestational age of 20 weeks detected a perineal mass, but other parameters were normal. He was born via spontaneous vaginal delivery after an uncomplicated pregnancy, at a gestational age of 38 weeks and his birth weight was 3,280g. The mother was healthy and did not use any medication during pregnancy nor in the year before pregnancy.

**Figure 1:**
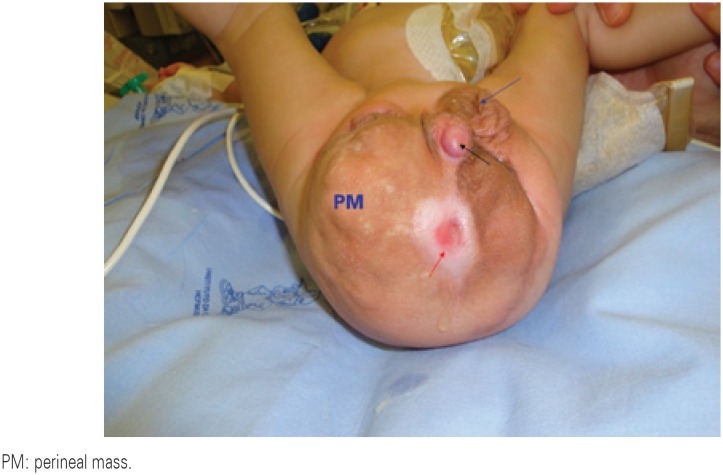
Epithelized perineal mass with cutaneous fistulization. Red arrow: fistulous orifice with urine output; black arrow: hipospadic urethral orifice; blue arrow: left scrotum

The child's physical examination revealed an epithelized perineal mass with cutaneous orifices which had urine fistulization, hipotrofic perineal musculature, bilateral congenital clubfoot, ambiguity of genitalia with a hipospadic urethra, criptorquidy bilateral with nonpalpable testis and imperforate anus (Figure 1).

**Figure 2:**
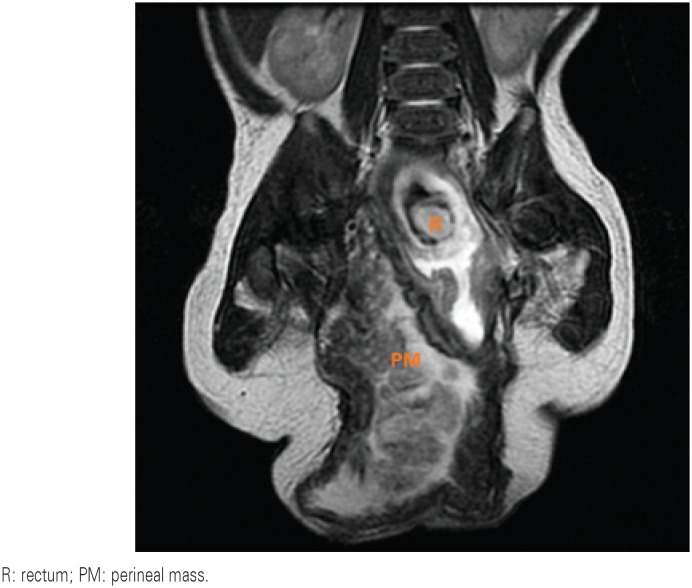
Magnetic resonance. Volumous perineal mass, extending superiorly to the right obturator, with very thick walls and heterogeneous content with neurovascular pedicle from the right sacral region

**Figure 3:**
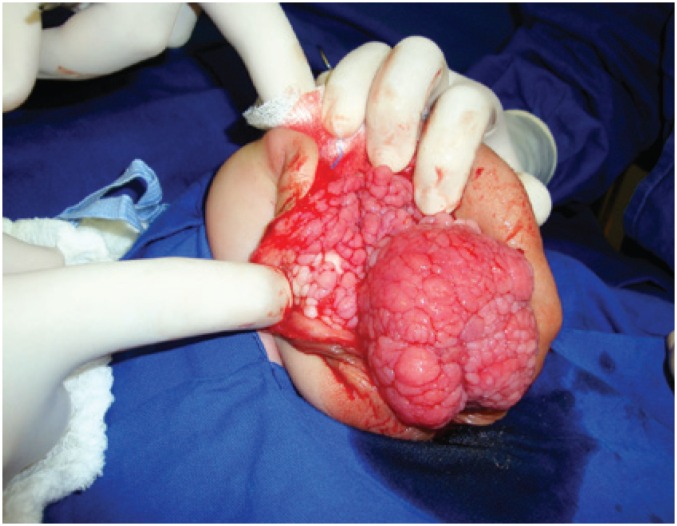
Lobulated mass after midline perineal incision

After birth, he presented fecal and urine content trough an urethral orifice and a colostomy was constructed. Subsequent laboratorial investigation showed karyotype 46 XY and normal ultrasound of urinary upper tract. The investigation with magnetic resonance showed a volumous perineal sac extending superiorly to the right obturator, with very thick walls and heterogeneous content with neurovascular pedicle from the right sacral region. In addition, we observed a slightly dilated rectosigmoid communicating with hernia sac, bladder with small repletion and elongated morphology with proeminent urachus. The urethra was poorly characterized, apparently continuing to a micropenis externalized outside of the hernia sac. Ureters had normal caliber. No structure was characterized as follows with their usual morphologies: uterus, ovaries, prostate and testicles (Figure 2). When the patient was 3 years old, he underwent perineal exploration through a midline perineal incision. The mass presented a lobulated aspect and was excised. Intraoperative finding was posterior bladder wall fistulization to a colon segment that was sutured (Figure 3). Both testicles were located at the inguinal canal and the orquidopexy was performed straightfoward. A Duplay neourethroplasty and a coloanal anastomosis were performed. The histopathological examination of the perineal mass revealed a hamartoma composed of fibrous connective tissue with proliferation of capilares and blood vessels, nerve bundles, fibroblasts, epithelium, smooth muscle and irregular ductal formations, covered by urothelial. No complications were seen in the surgery. After 16 months of follow-up, the patient was in fairly good condition. His lower limb neurological function was normal. However, the patient has a continuous urinary leakage and his bowel reconstruction must occur in the future.

## DISCUSSION

Association of a perineal hamartoma with anorectal anomaly is uncommon, and the additional presence of criptorquidy and congenital clubfoot turns our case extremely rare. Masses of the perineum in infants primarily consist of accessory or ectopic scrotum or perineal lipomas.^(2–4)^

Surgical excision of these lesions is generally straightforward. However, when the lesion occurs in a patient with an anorectal malformation and it is at the site where the future anus will be, surgical management may be more complex. Accurate pathological interpretation of the excised specimen is also of great importance.

A review with 2,000 cases of anorectal malformations revealed 22 cases with association to unusual perineal masses divided into 3 categories of lesions characterized as lipomas, vascular anomalies, and hamartomas/choristomas.^(1)^ In this review, eight cases of hamartomas were mentioned. All patients were girls and had a variety of different anorectal malformations. The typical lesion was a polypoid protuberance from the perineum at the site where the neoanus was reconstructed. A variety of unusual tissues such as nephrogenic rests, ectopic bone, and endocervical type mucosa were found in these lesions. One patient had initial treatment at a community hospital where the pathologist found perineal glandular tissue (endoderm).

In the present case, sacrococcygeal teratoma was a differential diagnosis. In the radiological study, sacrococcygeal teratomas present with soft tissue density and calcifications in 50% of cases (divided into amorphous, punctate and spiculated), all suggestive of benign tissue. On magnetic resonance imaging these tumors are characterized by hypointense signal on T1 and hyperintense in T2. Magnetic resonance image is considered the method of choice during the preoperative evaluation and it provides exact boundaries, topographic location, relationship to other pelvic organs and spine.

The association of hamartoma perineal with anorectal malformations is uncommon, and with genital abnormalities is extremely rare. Various hypotheses have been developed to explain the association between these pathologies.^(5)^ Stephens attempted to explain the embryogenesis of these anomalies including perineal anorectal malformations through a deformation by an abnormal pressure on the developing fetus. This pressure occurs in complicated pregnancies by oligohydramnios. The results of this pressure are skin dimples or joint contractures.^(6)^

The presentation of perineal mass with anorectal malformations, scrotal abnormalities and genital malformations, suggests possible growth of embryonic tissue structures of the phallus, from the urethral fold and genital, anal and perineal region that develop these events.^(6,7)^ It has been postulated that the normal development of the urogenital system and anorectum in human embryos may depend on the integrity of the mesenchymally-derived urorectal septum.^(5)^ Once the patient has an hipotrofic perineal musculature, both urinary and bowel continence may be affected. Because of the complexity of the case we report, long term results are difficult to predict.
